# Determination of Total Apigenin in Herbs by Micellar Electrokinetic Chromatography with UV Detection

**DOI:** 10.1155/2016/3827832

**Published:** 2016-06-29

**Authors:** Rafał Głowacki, Paulina Furmaniak, Paweł Kubalczyk, Kamila Borowczyk

**Affiliations:** Department of Environmental Chemistry, Faculty of Chemistry, University of Łódź, 163 Pomorska Street, 90-236 Łódź, Poland

## Abstract

Apigenin is a naturally occurring plant flavone that exhibits strong antioxidant, anti-inflammatory, and antitumor properties. A MEKC-UV based method was developed for the determination of total apigenin in selected herbs. Application of pseudostationary phase in the form of SDS micelles resulted in great repeatability of retention times and peak areas. A buffer solution consisting of 30 mmol/L sodium borate (pH 10.2), 10% acetonitrile, and 10 mmol/L sodium dodecyl sulfate was found to be the most suitable BGE for the separation. The method was validated and calibrated for total apigenin in the range of 1.0–100 *μ*mol/L (*R* ^2^ = 0.9994). The limits of detection and quantification were 0.48 *μ*mol/L and 0.92 *μ*mol/L, respectively. This precise and robust method was successfully applied to the analysis of plant samples for total apigenin content.

## 1. Introduction

**Figure 1:**
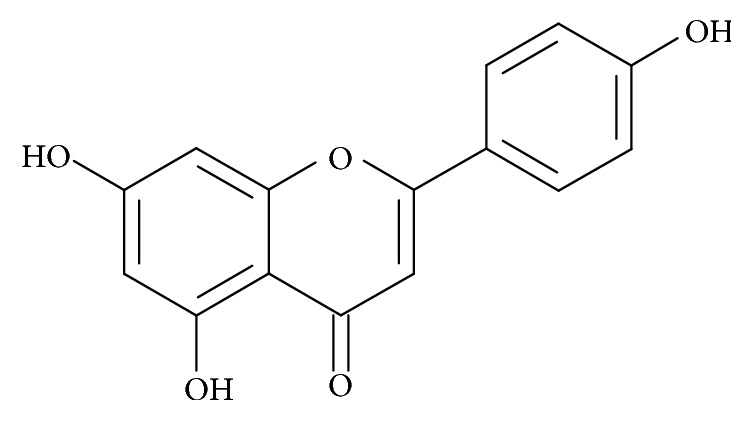
The chemical structure of the apigenin.

Flavonoids belong to the derivatives of 2-phenyl-benzo-*γ*-pyrone and are widespread in the superficial layers of the plant tissues. They are responsible for color of plants and protect them from harmful effects of fungi or insects. The presence of different functional groups in the molecules of flavonoids is responsible for their biological activity making them active components of the cellular metabolism [1]. It is also well established that flavonoids make a beneficial impact on human health [2, 3]. Taking into consideration differences in chemical structure, flavonoids can be divided into flavanones, flavanols, flavones, isoflavones, and flavonols. Generally they exist in two forms, namely, the aglycones and the *β*-glycosides [4]. Flavonols (kaempferol, quercetin) and flavones (apigenin, luteolin) occur most frequently in the form of glycosides and in such form are consumed by humans [5]. It is commonly known that herbs smell great and have a good influence on taste of cooked dishes. Moreover, interesting linkage between consumption of herbs rich in flavonoids and beneficial impact on some disorders has been observed. In living organisms apigenin (API), a naturally occurring plant flavone, acts as antioxidant and exhibits anti-inflammatory activities and prevents LDL oxidation as well as oxidation of vitamins C and E and glutathione [6]. API (Figure 1) possesses also antimutagenic and antiviral properties and inhibits the proliferation of various human cancer cells, including breast, cervical, lung, liver, prostate, gastric, and hematologic cancer cells [7, 8].

The well established importance of separation techniques has prompted elaboration of analytical methods enabling determination of biologically important compounds in different matrices. Herbs and aromatic plants such as* Petroselinum crispum* [9],* Rosmarinus* [10], and* Origanum vulgare* [11] have been studied for their biological activity. Among the numerous chromatographic techniques the use of capillary electrophoresis for the determination of flavonoids present in fruits and vegetables is now increasingly frequent. During the last decade a few electrophoretic methods have been reported for the determination of flavonoids in plant material. Şanli and Lunte determined flavonoids including API in* Chamomile* and* Linden* extracts with the use of capillary zone electrophoresis [12]. Boiteux et al. studied antimicrobial activity of plant extracts of chamomile, oregano, thyme, and creosote bush [13]. Honegr et al. used large-volume sample stacking with polarity switching for determination of natural polyphenols in* Orthosiphon stamineus Benth* [14]. Fonseca and Tavares determined free and total API in extracts of* Chamomilla recutita* [15]. All mentioned above methods show that the CE can be recognized as an alternative technique to HPLC. Moreover, contrary to liquid chromatography CE exhibits many advantages such as the low consumption of reagents and samples, short time of analysis, high resolution, and relatively low cost of operation. The main drawback of CE, when UV or FLD detectors are used, is a relatively high detection limit, which is the consequence of a short optical path length. Simple injection of a larger volume of a sample does not help since a long-injected zone is detrimental to separation efficiency and resolution. Despite many advances in electrophoretic techniques, a complex matrix cannot be injected directly into the capillary; thus an appropriate sample pretreatment is needed. Moreover, it is commonly known that the proper sample collection and preparation are the most difficult and time-consuming steps of the overall analytical procedure.

The goal of this study was to develop a simple and robust CE-UV based method for the analysis of some herbs for total API. Analytical conditions were optimized in order to determine the content of API in* Petroselinum crispum*,* Rosmarinus officinalis*,* Thymus vulgaris L*.,* Origanum vulgare*,* Origanum majorana L.*,* Salvia officinalis L.*, and* Levisticum officinale* by micellar electrokinetic chromatography (MEKC) with UV diode array detection. The assay allows determination of API with excellent reproducibility; thus it was possible to reach proper precision of the measurements. It is safe for the operator and environmentally friendly, meeting the needs of the biological sample analysis.

## 2. Experimental

### 2.1. Chemicals

All reagents and solvents were of either analytical grade or chromatographic purity. Apigenin (API), sodium tetraborate (Na_2_B_4_O_7_·10H_2_O), and sodium dodecyl sulfate (SDS) were obtained from Sigma Aldrich Company (St. Louis, MO, USA). HPLC grade acetonitrile (MeCN) and methanol (MeOH) were purchased from J. T. Baker (Deventer, Netherlands). Hydrochloric acid (HCl) and sodium hydroxide (NaOH) were purchased from POCH S.A. (Gliwice, Poland). The water used to prepare the solutions purified and deionized using a Millipore Milli-Q-RG System (Watford, UK). The pH of buffers was adjusted by potentiometric titrations. All solutions were prepared daily with purified water prior to use.

### 2.2. Apparatus

A Hewlett-Packard 3D Capillary Electrophoresis System (Waldbronn, Germany) with a UV-Vis absorbance diode array detector (DAD) was used. A fused-silica capillary with size 50 *μ*m ID × 60 cm (effective length 51.5 cm) was purchased from Polymicro Technologies (Phoenix, USA). For data handling and quantification HP ChemStation software was used. The pH was measured with a Crison Instruments BASIC 20 pH-meter (Barcelona, Spain). During experiments a Hettich Mikro 200R centrifuge with fast cool function (Hettich Zentrifugen, Tuttlingen, Germany) was used.

### 2.3. Electrophoretic Conditions

At the beginning of each day, the capillary was flushed with 0.1 mol/L NaOH solution for 5 min, deionized water for 5 min, and background electrolyte (BGE) for 15 min to permit equilibration. A mixture of 0.03 mol/L pH 10.2 sodium tetraborate, 0.01 mol/L sodium dodecyl sulfate (SDS), and 10% MeCN has acted as the BGE solution. Analyses were performed at 25°C and the detector was set to measure peaks at 390 nm. The electrophoresis system was operated under normal polarity and analyses were performed at the voltage of 30 kV. Samples were injected hydrodynamically for 30 s (50 mbar). Between runs the capillary was washed with 0.1 mol/L NaOH for 1 min, next water for 1 min, and finally fresh BGE for 5 min.

### 2.4. Sample Preparation

**Figure 2:**
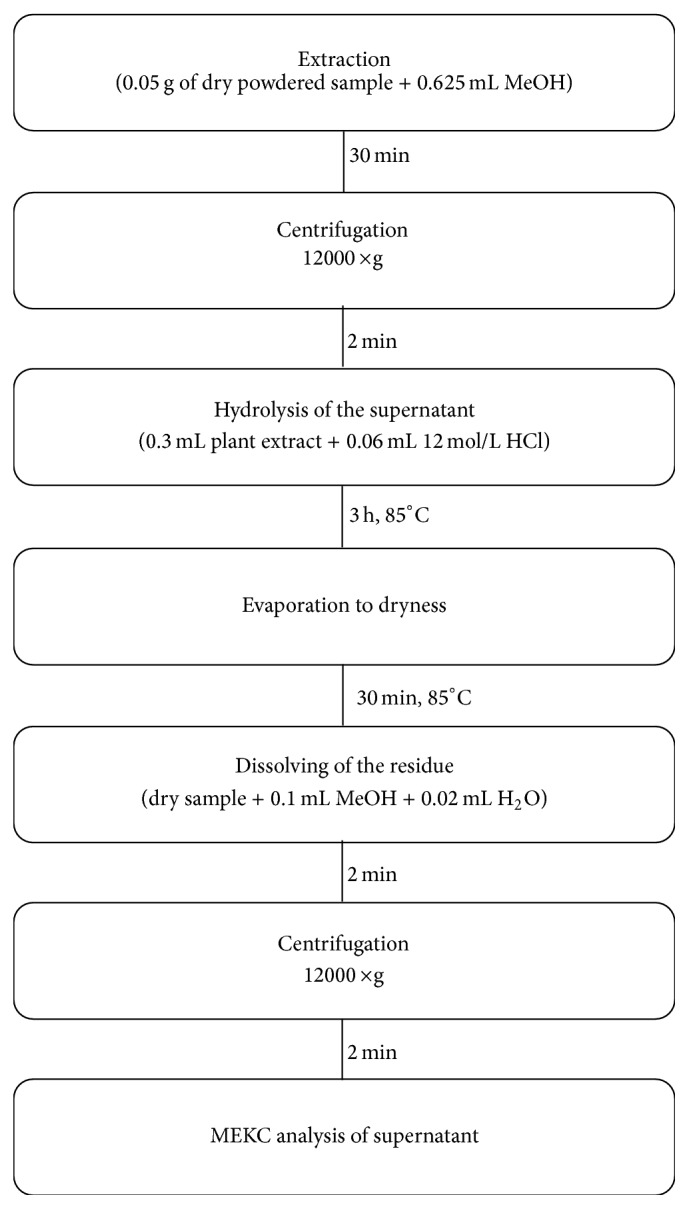
A flowchart diagram of sample preparation.

Dry samples of* Petroselinum crispum*,* Rosmarinus L.*,* Thymus vulgaris L.*,* Origanum vulgare*,* Origanum majorana L.*,* Salvia officinalis L.*, and* Levisticum officinale* were purchased from the local supermarkets. Fresh parsley leaves (*P. crispum*) were purchased from a local store (Lodz, Poland) and dried at 150°C for 30 min prior to use. Dry plant samples were then powdered in a mortar. It is well known that the addition of organic solvent (MeCN or MeOH) could improve API solubility. Portions of the powdered herbs (0.05 g) were extracted with 0.625 mL of MeOH at room temperature for 30 min followed by centrifugation (12000 ×g, 2 min). Then, 0.3 mL portions of the supernatant were collected in glass ampoules. The extracts were hydrolyzed in acidic conditions according to the modified procedure [16]. Briefly, to the methanolic extract 0.06 mL of 12 mol/L hydrochloric acid was added. In the case of parsley prior to hydrolysis a sample was diluted 10-fold with MeOH. The hydrolysis reaction was performed in sealed ampoule at 85°C. After 2 hours of hydrolysis ampoule was opened and evaporated to dryness at 85°C. The residue was dissolved in 0.1 mL of MeOH and 0.02 mL of deionized water was added. Then, the sample was mixed, centrifuged (12000 ×g, 5 min), and an aliquot of supernatant was injected into the capillary. A flowchart diagram of sample preparation is shown in Figure 2.

### 2.5. Calibration of the Method

A stock solution of 0.1 mol/L API needed in the method development procedure was prepared by dissolving appropriate amount of compound in 0.008 mol/L NaOH in MeOH. The working standard solutions were prepared by dilution with water as needed. For preparation of calibration standards, portions of 300 *μ*L of methanolic plant extracts were placed each in a sample tube and spiked with the growing amounts of working standard solution of API to provide a final API concentration from 1.0 to 100 *μ*mol/L. Calibration standards were prepared in five replicates. To the methanolic extract 0.06 mL of 12 mol/L hydrochloric acid was added. After hydrolysis (2 h, 85°C) ampoules were opened and evaporated to dryness at 85°C. Then, 0.1 mL of MeOH and 0.02 mL of deionized water were added to dissolve the residue. Next, the samples were mixed, centrifuged (12000 ×g, 5 min), and finally aliquots of supernatants were introduced into the capillary. Since small changes in migration times can result in peak area fluctuations corrected peak area was exploited during the study. The time corrected peak area was calculated by dividing the raw peak areas by their corresponding migration times. The corrected peak areas of API were plotted versus analyte concentration and curve was fitted by least-square linear regression analysis.

### 2.6. Validation

The method was validated according to the guidelines for biological sample analysis [17]. The repeatability of the measurements was tested by injecting standard solution in ten replicates. The precision and accuracy of the determination were accomplished by the analysis of parsley hydrolysate spiked with known amounts of API. Three concentrations representing the entire range of the calibration curve were studied: one near the limit of quantification (LOQ), one near the center, and one near the upper boundary of the standard curve. Measured concentrations were assessed by the application of calibration curve obtained on that occasion. Precision is expressed in terms of relative standard deviation, whereas accuracy as the percentage of analyte recovery is calculated by expressing the mean measured amount as percentage of added amount.

Accuracy was calculated with the use of formula:

(1) Recovery %=measured  amount−endogenous  contentadded  amount×100%.

The linearity of the method was tested using seven-point calibration plot, and at each concentration five replicates were assayed. The interval of linear response of the detector with respect to API covered the concentration range from 1.0 to 100 *μ*mol/L.

## 3. Results and Discussion

### 3.1. Separation Optimization

#### 3.1.1. Effect of pH and the Buffer Concentration

The physicochemical properties of analyte as well as BGE play a crucial role during the electrophoretic process. The pH and concentration of BGE were altered to affect changes in migration and selectivity of the separation, primarily by changing electrokinetic velocity. The BGE was borate buffer with addition of 10% MeCN and 10 mmol/L sodium dodecyl sulfate (SDS). The capillary was conditioned with a mixture of BGE and SDS; then the sample was hydrodynamically injected into the capillary. To optimize separation conditions BGE pH of 9.0, 9.6, 9.8, 10.0, 10.2, 10.4, and 10.8 was assayed. When pH is greater than 9.0 the phenolic hydroxyl groups of API are dissociated to phenolate anions which exhibit higher affinity to the anodic end of the capillary than unionized form. In the case of much more negatively charged micelles this effect is stronger but is compensated by influence of the size of the molecule. However, under these conditions the EOF moves both anions to the cathode. Application of the pH values lower than 9.0 resulted in a weak separation and substantial enlargement of the analysis time.

**Figure 3:**
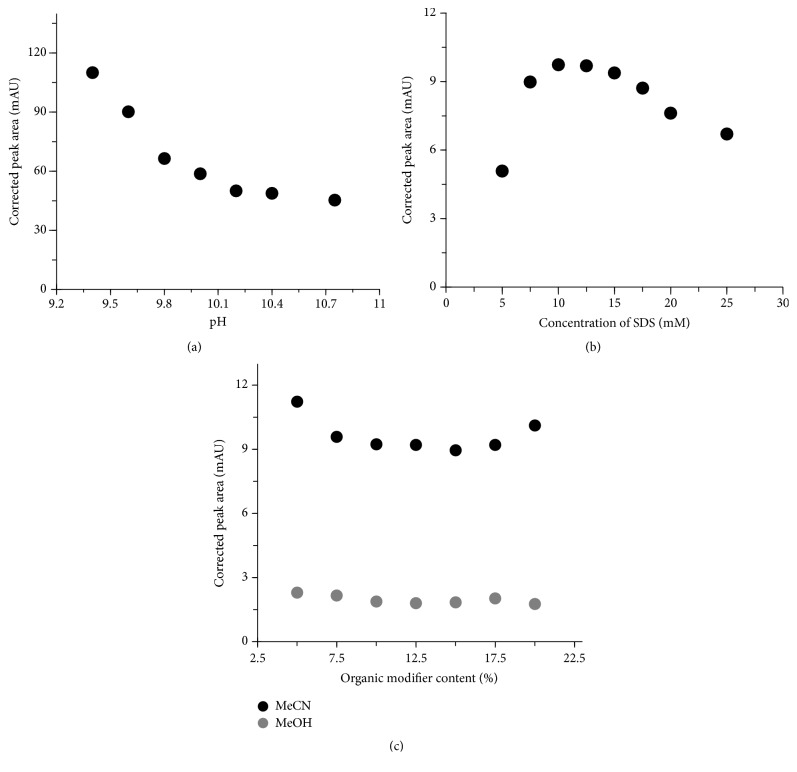
The influence of pH of BGE (a), SDS concentration (b), and organic modifier content (c) on peak area. Other separation conditions are described in Section 2.3.

Borate buffer is often employed in analyses of flavonoids due to its possibility of creating anionic complexes with compounds possessing neighboring hydroxyl groups such as luteolin, API, or quercetin [14]. During the optimization of borate buffer concentration the MeCN and sodium dodecyl sulfate (SDS) amounted to 10% (v/v) and 10 mmol/L, respectively. With increasing concentration of the buffer a quality of separation was increased, but the time of migration was extended (data not shown). Moreover, as can be seen in Figure 3(a) the corrected peak area significantly decreases with increasing pH. Taking into consideration separation time as well as the resolution, selectivity, and the peak symmetry, 0.03 mol/L pH 10.2 borate buffer was chosen for the further experiments.

#### 3.1.2. Effect of SDS Concentration

In MEKC a surfactant is added to BGE used in capillary zone electrophoresis in the amount that is sufficient to create micelles. In our research we used anionic surfactant SDS, which efficiently generates pseudostationary phase. Despite the fact that the increase of SDS significantly extended analysis time, a high concentration of micelles resulted in better separation. Micelles formed from anionic SDS migrate towards the anode (in the direction opposite to the EOF). Since the EOF is generally higher than the migration of micelles at pH 10.2, the net movement is in direction of EOF. In a homogeneous electric field, with the normal polarization, micelles permeate into a sample zone sweeping the analytes into a narrow zone resulting in LOD improvement. Then analytes interact with micelles and are separated according to MEKC mechanism. Eight compositions of BGE containing 0.03 mol/L pH 10.2 borate buffer, 10% of MeCN, and different SDS concentrations were tested to study the effect of SDS content on separation efficiency. We have found that SDS concentration ranged from 5 to 25.0 mmol/L significantly influences corrected peak area (Figure 3(b)). Importantly, each concentration tested allowed good separation of API from unknown sample components. Taking into consideration the analysis time as well as quality of the separation for further analysis the concentration of 10.0 mmol/L SDS was chosen.

#### 3.1.3. Influence of Organic Modifier

Organic modifier plays an important role in a MEKC separation, mainly by hydrophobicity and viscosity changing. Indeed, the prolongation of the analysis time due to the presence of MeCN/MeOH is associated with the inhibitory effect of organic solvent on the viscosity and dielectric constant. Seven different organic modifier compositions amounting to 5, 7.5, 10, 12.5, 15, 17.5, and 20% were assayed. Finally, in the case of MeCN almost fourfold signal enhancement compared to MeOH was obtained (Figure 3(c)). Thus, for further analysis 10% of MeCN was chosen. Extension of migration time concomitant with the increase of MeCN/MeOH content was also observed (data not shown).

**Figure 4:**
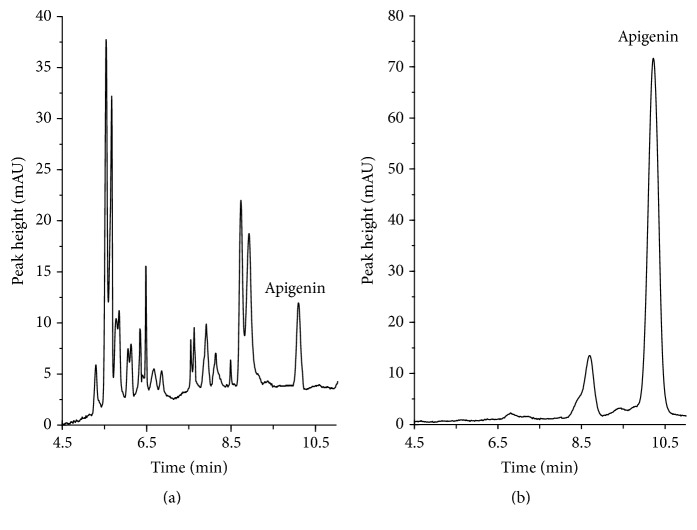
Representative chromatograms of oregano (a) and parsley leaves (b), assayed according to the procedure described in Section 2.4. Electrophoretical conditions are described in Section 2.3.

Additional studies were carried out to establish optimal conditions for API separation. The volume of sample injection and the separation voltage were tested (data not shown). Making allowance for all tested factors, such as MeCN content, pH, and concentration of BGE as well as SDS concentration, the best results were obtained for the electrolyte that consisted of 0.03 mol/L pH 10.2 borate buffer with 10% (v/v) of MeCN and 0.01 mmol/L SDS.  Figure 4 shows representative electropherograms of the herbs after extraction and hydrolysis under the optimum conditions.

### 3.2. Sample Preparation

The selection of optimal separation conditions depends on the specificity of analyzed sample. Despite the huge progress in separation techniques, a complex matrix cannot be injected directly into the analytical instrument. Sample pretreatment is required to remove the matrix components which could interfere with an analyte and deteriorate quality of separation or detection. Sample pretreatment is frequently used to preconcentrate the analytes of interest from the target matrices. Some types of samples such as water and other fluids are suitable for relatively simple collection and preparation. Solid samples, including fruits, plants, or vegetables, require physical homogenization and more sophisticated pretreatment. Several methods have been developed for the determination of flavonoids in plant samples [18–20]. Different sample preparation approaches have been also used [21–23]. These methods utilize hydrolysis and extraction techniques such as fluid extraction, pressurized liquid extraction, ultrasonic bath extraction, or extraction with the use of Soxhlet apparatus. In all above-mentioned cases extraction led to methoxyoxaloyl group removal from the conjugates. In the next step hydrolysis was aimed at breaking the bonds between *β*-aglycones and sugar molecules.

**Figure 5:**
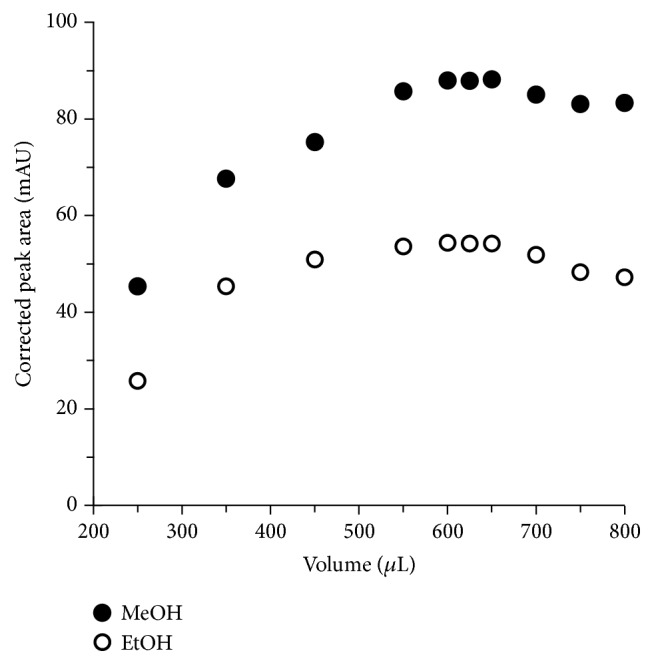
The influence of volume and type of solvent on extraction yield (expressed as corrected peak area).

**Figure 6:**
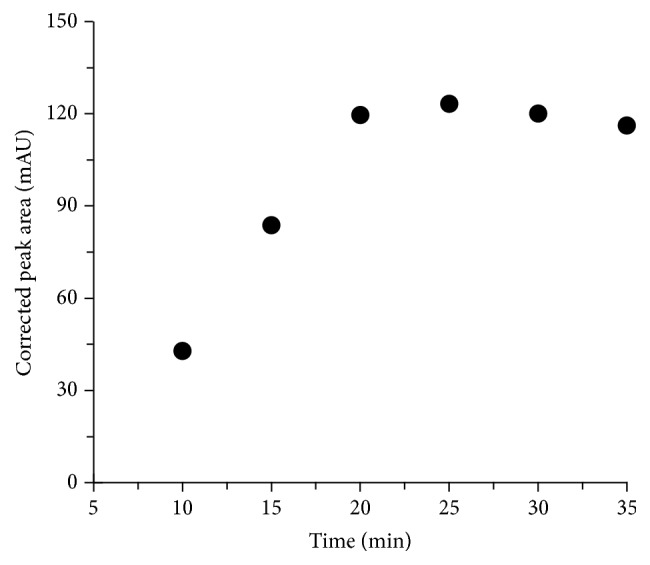
The influence of the time on extraction yield (expressed as corrected peak area).

It is commonly known that solid tissues must be shredded and homogenized before the analysis by liquid phase separation techniques. In our approach sample preparation development consisted of establishing optimal recovery of API from the standard solution and then transferring those conditions for use with homogenate of herb samples. The extraction and hydrolysis efficiencies were estimated electrophoretically by comparison of API corrected peak areas. Sample preparation method included homogenization, liquid extraction, and acid hydrolysis, followed by dissolution of the residue in the mixture of MeOH/H_2_O. The influence of the type and volume of the solvent as well as extraction time on method precision has been studied. It has been shown that MeOH is more suitable for API extraction than EtOH. Moreover, as is evident in Figure 5 the quantity of MeOH strongly affects the extraction yield (expressed as corrected peak area). As can be seen from Figure 6 time of extraction significantly affects extraction yield. The signal area of each API extract was compared with a standard API sample. The efficiency of the process was calculated using the following formula: *E*_eff_ = (extracted amount of “a”/original amount of “a”) × 100%. Since concentration of API in herbs is substantial, yield of extraction is not a problem. A major issue concerns its reproducibility. Recovery of the analyte from the herb samples in our approach was relatively good and amounts to 68.5 ± 4.0%. Some investigators obtained similar results (72.7%–89.5%) for API determination in celery [24]. In most cases extraction yield was much better and amounted to 94.4%–97.2% for baicalin determination in herbs [25], 96.7% for flavonoids in yellow toadflax herb [26], and 98.2% for flavonoids in* Gingko biloba* leaves [27]. Importantly, recovery in this method was highly reproducible, making procedure useful for API determination.

After extraction the samples were hydrolyzed with the use of HCl and then evaporated to dryness. The residue was dissolved in the mixture of MeOH/H_2_O. Lower viscosity of the sample makes measurements simpler and more reliable; thus several solvent systems (1 : 1, 1 : 2, 1 : 3, 1 : 4, 1 : 5, and 1 : 6) were tested.

It should be emphasized that sample dilution is advantageous for error decrease; on the other hand it causes inferior limits of detection and quantification. However, amount of API in herbs is substantial; thus finally for the residue dissolution we decided to use 0.12 mL of the mixture that consisted of 0.1 mL MeOH and 0.02 mL H_2_O. The addition of water resulted in better solubility of the hydrolysate whereas the total error of the method did not exceed 7.9%.

### 3.3. Validation of the Method

The RSD value for migration time of API was 1.09%, whereas the RSD value of corrected peak area did not exceed 5.7%.

The calculated lowest and highest values for the precision and accuracy of the method were from 5.8 to 12.1% and from 96.1 to 107.1%, respectively.

The described method showed good linearity between corrected peak area and concentration of API in all herb samples. The equation for the linear regression for API was *y* = 0.4098*x* + 55.249 (*R* ^2^ = 0.9994). Interassay and intra-assay variation were 7.6% and 4.9%, respectively.

The LOQ defined as the concentration that produced a detector signal that could be clearly distinguished from the baseline (higher than ninefold noise level of baseline) was 0.92 *μ*mol/L (0.25 *μ*g/mL). The limit of detection (LOD) based on the signal-to-noise ratio equal to 3 was set at the level of 0.48 *μ*mol/L (0.13 *μ*g/mL). The LOQ and LOD are low and allowed the analysis of all the samples studied. Other validation parameters of fitted calibration curve were very good as can be seen in Table 1. Fonseca and Tavares presented a method for the determination of API in* Chamomile recutita* where LOD was 3.8 *μ*g/mL [15]. Peng et al. have described electrophoretic method for the determination of active components in rosemary where LOD value was 2.0 *μ*g/mL with an analysis time of 24 minutes [28]. Şanli and Lunte have proposed an assay based on CZE-UV with LOQ equal to 0.5 *μ*g/mL [12]. Jiang et al. published methodology enabled determination of API in* Paulownia tomentosa *with significantly higher LOD equal to 5 *μ*g/mL [29].

### 3.4. Application of the Method

The final phase of the study involved implementation of the optimized and validated MEKC method for the analysis of herb samples. API was determined in seven selected commercially available herbs. The identification of peaks in the plant hydrolysates was made by comparison of migration times in real and standard samples. The signals were also identified by comparison of UV spectra of the analyte. We have found that the highest content of API occurred in parsley leaves (137.7 mg/g of dry sample). Content of API in the fresh parsley leaves purchased from the supermarket and dried under laboratory conditions was similar to these observed by other investigators [5, 30]. API contents in oregano (14.0 mg/g of dry sample) and marjoram (2.5 mg/g of dry sample) were also high, and the results obtained are comparable to those described earlier [31, 32]. The analytical results are summarized in Table 2. Obtained results clearly indicate that elaborated assay can be successfully utilized for API determination in plant samples.

## 4. Conclusions

A quick, simple, and robust method for the determination of API in herbs has been developed. It is commonly known that capillary electrophoresis methods suffer from poor reproducibility of migration times, especially when biological samples are analyzed. The big advance in our approach is the application of pseudostationary phase in the form of SDS micelles that resulted in great repeatability of retention times and peak areas. Application of sweeping MEKC technique for signal enhancement is also advantageous. Our assay exhibits about 40-fold lower LOD in comparison with earlier published MEKC procedure [29]. The studies have confirmed that besides separation conditions also sample preparation makes inroads upon quality of the results. The assay yielded high analyte recovery with excellent reproducibility; thus it was possible to reach proper precision of the measurements. The method was successfully applied for the analysis of seven commonplace herbs. It is safe for the operator and environmentally friendly by using really small amounts of organic solvents, meeting the needs of the biological sample analysis.

## Figures and Tables

**Table 1:** Validation data.

Sample	Amount added^(a)^ (nmol/mL)	Amount found ± SD (nmol/mL)	RSD (%)	Recovery (%)
Extract	0	137.7 ± 15.1^(b)^	5.8	—
	1	1.1 ± 0.1	12.1	107.1
	50	48.0 ± 5.1	10.6	96.1
	100	100.3 ± 9.5	9.5	100.3

^(a)^ *n* = 5.

^(b)^Endogenous concentration.

**Table 2:** Concentration of apigenin in different herbs.

Herb	Average value ± SD	
	(*µ*mol/mL)	(mg/g)
*Petroselinum crispum*	122.4 ± 13.4^a^	137.7 ± 15.1^a^
	112.0 ± 12.2^b^	126.0 ± 13.7^b^
*Rosmarinus L.*	0.5 ± 0.2^c^	0.5 ± 0.2^c^
*Thymus vulgaris L.*	0.7 ± 0.3^c^	0.7 ± 0.3^c^
*Origanum vulgare*	12.4 ± 2.7	14.0 ± 2.7
*Origanum majorana L.*	2.2 ± 0.4	2.5 ± 0.5
*Salvia officinalis L. *	1.0 ± 0.6	1.1 ± 0.6
*Levisticum*	1.3 ± 0.6	1.4 ± 0.7

^a^Parsley bought from the supermarket, fresh leaves.

^b^Parsley bought from a local store.

^c^Below limit of quantification.
